# Conformationally Altered C-Reactive Protein Capable of Binding to Atherogenic Lipoproteins Reduces Atherosclerosis

**DOI:** 10.3389/fimmu.2020.01780

**Published:** 2020-08-11

**Authors:** Asmita Pathak, Sanjay K. Singh, Douglas P. Thewke, Alok Agrawal

**Affiliations:** Department of Biomedical Sciences, James H. Quillen College of Medicine, East Tennessee State University, Johnson City, TN, United States

**Keywords:** atherosclerosis, C-reactive protein, inflammation, low-density lipoprotein, phosphocholine

## Abstract

The aim of this study was to test the hypothesis that C-reactive protein (CRP) protects against the development of atherosclerosis and that a conformational alteration of wild-type CRP is necessary for CRP to do so. Atherosclerosis is an inflammatory cardiovascular disease and CRP is a plasma protein produced by the liver in inflammatory states. The co-localization of CRP and low-density lipoproteins (LDL) at atherosclerotic lesions suggests a possible role of CRP in atherosclerosis. CRP binds to phosphocholine-containing molecules but does not interact with LDL unless the phosphocholine groups in LDL are exposed. However, CRP can bind to LDL, without the exposure of phosphocholine groups, if the native conformation of CRP is altered. Previously, we reported a CRP mutant, F66A/T76Y/E81A, generated by site-directed mutagenesis, that did not bind to phosphocholine. Unexpectedly, this mutant CRP, without any more conformational alteration, was found to bind to atherogenic LDL. We hypothesized that this CRP mutant, unlike wild-type CRP, could be anti-atherosclerotic and, accordingly, the effects of mutant CRP on atherosclerosis in atherosclerosis-prone LDL receptor-deficient mice were evaluated. Administration of mutant CRP into mice every other day for a few weeks slowed the progression of atherosclerosis. The size of atherosclerotic lesions in the aorta of mice treated with mutant CRP for 9 weeks was ~40% smaller than the lesions in the aorta of untreated mice. Thus, mutant CRP conferred protection against atherosclerosis, providing a proof of concept that a local inflammation-induced structural change in wild-type CRP is a prerequisite for CRP to control the development of atherosclerosis.

## Introduction

Atherosclerosis is a chronic inflammatory disease whose development begins with the dysfunction of the endothelium of the arteries. Endothelial dysfunction leads to infiltration of plasma low-density lipoprotein (LDL) and monocytes in the arterial wall. LDL is subsequently deposited, modified and engulfed by monocyte-derived macrophages. Lipid-laden macrophage foam cells are proinflammatory which enhances the process of the formation of atherosclerotic lesions (1, 2). The inflammatory microenvironment of atherosclerotic lesions is characterized by macrophage activation, hypoxia, and lactate and proton generation, resulting in an acidic extracellular pH (3–7).

C-reactive protein (CRP) is a plasma protein produced by the liver in inflammatory states (8). CRP is also present, co-localized with modified LDL and macrophages, at atherosclerotic lesions in both humans and experimental animals (9, 10). CRP is composed of five identical subunits arranged in a pentameric symmetry. The molecular weight of each subunit is ~23 kDa and there are 206 amino acid residues in each subunit. CRP binds to molecules with exposed phosphocholine (PCh) groups in a Ca^2+^-dependent manner. There are five PCh-binding sites in the CRP pentamer, one site per subunit (11, 12).

Oxidized LDL (ox-LDL), enzymatically-modified LDL (E-LDL) and acetylated LDL (ac-LDL) are different forms of modified atherogenic LDL that are used in *in vitro* experiments (1, 2, 13). In the presence of Ca^2+^, native or recombinant wild-type (WT) CRP interacts with E-LDL due to the exposure of PCh groups on E-LDL, but does not interact with ox-LDL and ac-LDL (14–16). In the absence of Ca^2+^, WT CRP does not interact with any form of atherogenic LDL (16–19). The native pentameric structure of CRP is altered in response to a variety of experimental conditions (16–23). It has been shown that the pentameric structure of WT CRP is subtly altered by acidic pH and by oxidation, and conformationally altered CRP is capable of binding to atherogenic LDL independent of the PCh-binding site (17–19). Employing E-LDL, it has also been shown that if CRP is bound to atherogenic LDL, it prevents the formation of lipid-laden macrophage foam cells (24). Due to the presence of CRP at atherosclerotic lesions and due to the binding capability of CRP for atherogenic LDL under certain conditions, CRP has been implicated in the development of atherosclerosis (25).

We have previously reported a CRP mutant, F66A/T76Y/E81A, in which Phe^66^, Thr^76^, and Glu^81^ (three critical amino acid residues in the PCh-binding site) were substituted with Ala, Tyr, and Ala, respectively, to abolish the PCh-binding activity of CRP (26). Mutant CRP did not bind to PCh and was used as a tool to investigate the importance of the PCh-binding site in CRP-mediated protection of mice against pneumococcal infection (26). Biochemical characterization of this mutant CRP, in comparison to that of WT CRP, has been published previously (26). The overall structure of mutant CRP was pentameric and the mutation did not affect the stability of the protein *in vivo*. Mutant CRP circulated freely in the mouse serum and its rate of clearance *in vivo* was similar to that of WT CRP (26).

Further analysis of F66A/T76Y/E81A mutant CRP, presented here, revealed that mutant CRP had inadvertently gained the ability to bind to any protein that was immobilized on microtiter plates, including atherogenic forms of LDL, without the need for any further inflammatory milieu-dependent or acidic pH-induced structural change. We, therefore, hypothesized that mutant CRP might show an atheroprotective effect in murine models of atherosclerosis. We reasoned that even if there was no acidic pH around injected mutant CRP in the available murine models, mutant CRP would be able to recognize and bind atherogenic LDL and exert a protective effect on the development of atherosclerosis. Accordingly, in this study, we evaluated the effects of mutant CRP (F66A/T76Y/E81A) on the development of atherosclerosis employing LDL receptor-deficient (*Ldlr*^−/−^) mice, an animal model commonly used to investigate molecules involved in human atherosclerosis.

## Materials and Methods

### Preparation of F66A/T76Y/E81A Mutant CRP

The construction of F66A/T76Y/E81A mutant CRP cDNA used in this study has been reported previously (26). Mutant CRP was expressed in CHO cells using the ExpiCHO Expression System (Thermo Fisher Scientific), according to manufacturer's instructions. As described previously (26), mutant CRP was purified from cell culture supernatant by Ca^2+^-dependent affinity chromatography on a phosphoethanolamine-Sepharose column, since this CRP mutant does not bind to PCh. Mutant CRP was further purified by gel filtration on a Superose12 column. Eluted mutant CRP was immediately dialyzed against 10 mM Tris-HCl, 150 mM NaCl, pH 7.2 (TBS), containing 2 mM CaCl_2_, stored at 4°C, and was used within a week. The purity of CRP was confirmed by using denaturing SDS-PAGE. For *in vivo* experiments, purified mutant CRP was treated with the Detoxi-Gel Endotoxin Removing Gel (Thermo Fisher Scientific) according to manufacturer's instructions. The removal of endotoxin from mutant CRP preparations was confirmed by using the Limulus Amebocyte Lysate kit QCL-1000 (Lonza) according to manufacturer's instructions.

### Solid Phase Ligand-Binding Assay

The solid phase ligand-binding assay was used to determine the binding of mutant CRP to immobilized proteins, as described earlier (17–19). Ox-LDL, E-LDL, ac-LDL, factor H (Complement Technology) and amyloid β peptide 1-42 (Bachem, H-1368) were used as protein ligands. Ox-LDL, E-LDL, and ac-LDL were prepared as described previously (17–19, 24). Briefly, microtiter wells were coated with protein ligands (10 μg/ml) diluted in TBS and incubated overnight at 4°C. The unreacted sites in the wells were blocked with TBS containing 0.5% gelatin. Freshly purified mutant CRP was diluted in TBS-Ca (TBS containing 2 mM CaCl_2_, 0.1% gelatin and 0.02% Tween 20), added to the wells, and incubated overnight at 4°C. Bound mutant CRP was detected by using a polyclonal rabbit anti-human CRP antibody (Millipore Sigma, 235752). HRP-conjugated donkey anti-rabbit IgG (GE Healthcare) was used as the secondary antibody. Color was developed using ABTS as the substrate and the OD_405_ was read in a plate reader.

### Atherosclerosis Protection Experiments

**Figure 1:**
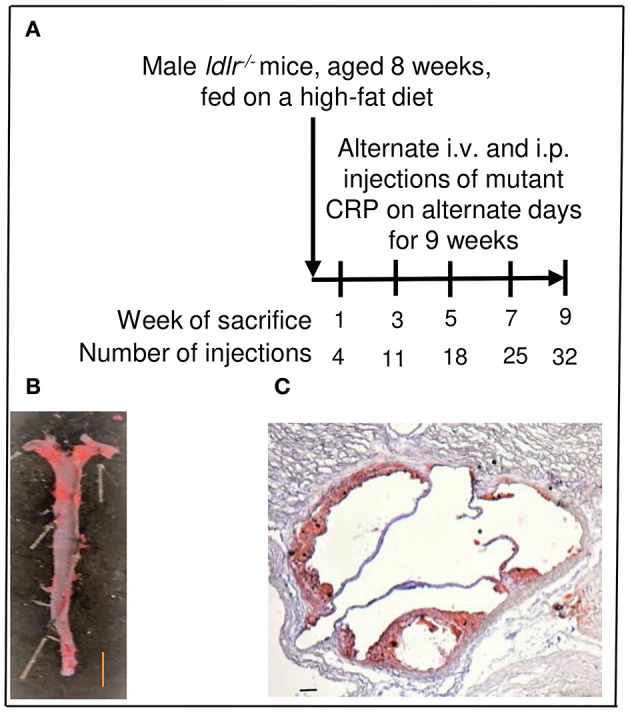
Assessment of atherosclerotic lesions in *ldlr*^−/−^ mice. **(A)** Protocol for administration of mutant CRP and for sacrifice of mice after mutant CRP administration. **(B)** Quantitation of atherosclerotic lesions in the aorta (*en face*). A representative Sudan IV-stained aorta is shown. The red colored areas are the lesions. Scale bar, 5 mm. **(C)** Quantitation of atherosclerotic lesions in the aortic root. A representative Oil Red O-stained aortic root section is shown. The red colored areas are the lesions. Scale bar, 100 μm.

Eight-week-old C57BL/6 male *ldlr*^−/−^ mice (Jackson Lab, 002207) were used in experiments according to protocols approved by and conducted in accordance with the guidelines administered by the Institutional Animal Care and Usage Committee of East Tennessee State University. Sixty mice were fed on a high-fat western-type diet (21% fat, 0.2% cholesterol), purchased from Envigo (TD.88137), for 10 weeks. After 1 week on high-fat diet, mice were divided into two groups of 30 mice in each group: untreated group and mutant CRP-treated group. Mice were injected with either TBS (untreated group) or mutant CRP (50 μg/injection) on alternate days for 9 weeks, via alternating intravenous and intraperitoneal routes (Figure 1A). Blood, heart and aorta were collected at five different time points (weeks 1, 3, 5, 7, and 9), as described previously (27). Briefly, six mice from each group, at each time point, were sacrificed, blood was collected by cardiac puncture, and the plasma was separated and stored frozen. After collecting blood, the heart was perfused with 10% formalin, followed by removing fat from the entire aorta. Heart was excised from the aorta, was cut into two halves, and the upper half with the aortic root was mounted using OCT medium and stored at −80°C. Following heart excision, aorta was excised and stored in 10% formalin at 4°C. The whole experiment was repeated once more employing another 60 mice and a separate, freshly purified batch of mutant CRP.

### Measurement of the Size of Atherosclerotic Lesions

To measure the size of atherosclerotic lesions in the whole aorta (*en face*), aorta was cut open longitudinally, stained with Sudan IV, and digitally photographed, as described previously (27). A total of 120 aortae (60 mice for untreated group and 60 mice for mutant CRP-treated group; 12 mice per time point) were processed, stained and photographed. A representative Sudan IV-stained aorta is shown in Figure 1B.

To measure the size of atherosclerotic lesions in the aortic root, 8 μm cross-sections of frozen OCT-embedded heart were collected from the appearance of the aortic valve leaflets to their disappearance (48–72 sections per heart). Every other cross-section of the entire aortic root was stained with Oil Red O for lipids, counterstained with hematoxylin, and digitally photographed, as described previously (27). A total of 120 hearts (60 mice for untreated group and 60 mice for mutant CRP-treated group; 12 mice per time point) were processed. A representative Oil Red O-stained aortic root section is shown in Figure 1C.

Digital photographs were acquired with an Olympus BX41 microscope equipped with a CCD color camera (QImaging). The stained lesion areas were quantified in digital images using the ImageJ software (28). Quantification of the lesion areas in the photographs was performed by two observers blinded to the experimental protocol. Non-parametric test (Mann-Whitney test) using GraphPad Prism software was employed to calculate the *p*-values.

### Immunostaining of CRP

Sudan IV-stained aorta was first processed to remove the stain, as described earlier (29). Immunostaining of CRP was performed using Vectastain ABC Elite kit (Vector laboratories, PK-6100) according to manufacturer's instructions. CRP was detected by using a polyclonal rabbit anti-human CRP antibody (Millipore Sigma, 235752). Biotinylated goat-anti rabbit IgG was used as the secondary antibody. Color was developed using DAB (Vector laboratories, ImmPACT DAB, SK-4105) as the substrate, according to manufacturer's instructions.

### Measurement of Lipoproteins in the Plasma

The concentrations of high-density lipoprotein (HDL) and LDL in the plasma were measured using Cholesterol Assay Kit-HDL and LDL/VLDL (Abcam; ab65390) according to manufacturer's instructions. Lipoprotein levels were measured in the pooled plasma samples collected at weeks 1, 3, 5, 7, and 9 (12 mice per time point). Unpaired student *t*-test was employed to calculate the *p*-values.

## Results

All experiments were performed three times, unless otherwise mentioned, and comparable results were obtained each time. Results of a representative experiment are shown in the figures where the raw data (OD_405_) were used to plot the curves.

### Mutant CRP Binds to Atherogenic LDL

**Figure 2:**
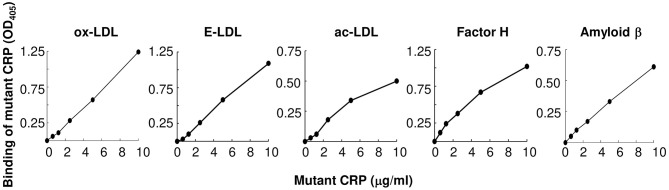
Binding of F66A/T76Y/E81A mutant CRP to immobilized proteins including atherogenic LDL at physiological pH. Microtiter wells were coated with protein ligands as shown. After blocking the unreacted sites in the wells, mutant CRP diluted in TBS-Ca was added to the wells and incubated for 2 h at 37°C. Bound mutant CRP was detected by using a rabbit anti-human CRP antibody and HRP-conjugated donkey anti-rabbit IgG. Color was developed and the OD was read at 405 nm. A representative of three experiments are shown.

As shown in Figure 2, mutant CRP bound to all three forms of atherogenic LDL immobilized on microtiter wells. The binding occurred at physiological conditions, that is, in the absence of any protein structure-modifying agent, such as acidic pH. Since mutant CRP does not bind to PCh (26), the binding of mutant CRP to either ox-LDL, E-LDL, or ac-LDL was independent of the PCh groups present in atherogenic LDL. In addition to testing the binding of mutant CRP to atherogenic LDL, we also included two other proteins, factor H and amyloid β peptide, in the binding assay. Mutant CRP also bound to factor H and amyloid β peptide in a concentration-dependent manner, similar to its binding to atherogenic LDL. These results suggest that mutant CRP did not recognize immobilized atherogenic LDL *per se*, but it recognized a pattern, as yet undefined, on immobilized proteins in general.

### Mutant CRP Reduces Atherosclerotic Lesions in the Whole Aorta

**Figure 3:**
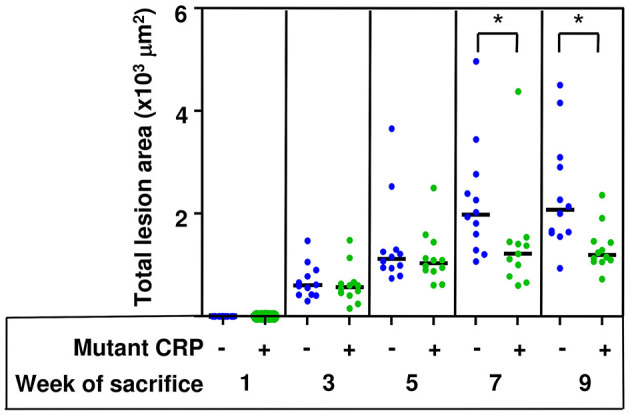
Reduction in the size of atherosclerotic lesions in the aorta of mutant CRP-treated mice. The scatter plot shows the total atherosclerotic lesion area in *en face* aorta from untreated and mutant CRP-treated mice. Data were collected at five different time points: 1, 3, 5, 7, and 9 weeks after mutant CRP administration (12 mice per group per time point). Each blue dot represents one mouse from the untreated group and each green dot represents one mouse from the mutant CRP-treated group. Horizontal black lines indicate the median total lesion area for each group. Asterisks denote statistically significant difference between untreated and mutant CRP-treated groups (*p ≤ 0.01).

The total size of all lesion areas in the whole aorta (*en face*) was measured. Figure 3 shows the combined results of two separate experiments with 12 mice in each group, for each time point. There was no effect of mutant CRP on the *en face* lesions for the first 5 weeks. The effect of mutant CRP on the lesion size was visible once the disease had progressed further. In comparison to untreated mice, the lesion area in mutant CRP-treated mice was 30.9% less after 7 weeks and 42% less after 9 weeks of CRP administration. As shown, in mutant CRP-treated mice, the lesion area did not increase after 5 weeks unlike in the untreated group where the lesion area kept increasing for another 2 weeks. Similar results were seen when the data from each of the two experiments (6 mice/group/time point/experiment) were analyzed separately (Supplemental Figure 1). We did not determine the specific stages of atherosclerosis at any time point.

### The Effects of Mutant CRP Are Not Visible at the Aortic Root Lesions

**Figure 4:**
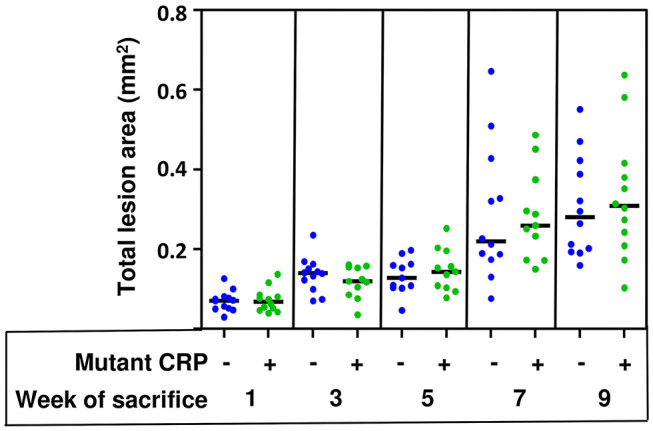
No reduction in the size of atherosclerotic lesions in the aortic root of mutant CRP-treated mice. The scatter plot shows the total atherosclerotic lesion area in aortic root sections from untreated and mutant CRP-treated mice. Data were collected at five different time points: 1, 3, 5, 7, and 9 weeks after mutant CRP administration (12 mice per group per time point). Each blue dot represents one mouse from the untreated group and each green dot represents one mouse from the mutant CRP-treated group. Horizontal black lines indicate the median total aortic root lesion area for each group.

The total size of all lesion areas in the aortic root was measured. Figure 4 shows the combined results of two separate experiments using 12 mice in each group, for each time point. As shown, the disease progressed for the entire duration of 9 weeks in untreated mice. However, the administration of mutant CRP did not affect the lesion size at any time point. There was no statistically significant difference in the lesion size at any time point between untreated and mutant CRP-treated groups of mice. Similar results were seen when the data from each of the two experiments (6 mice/group/time point/experiment) were analyzed separately (Supplemental Figure 2).

### Administered Mutant CRP Is Present in the Aorta

**Figure 5:**
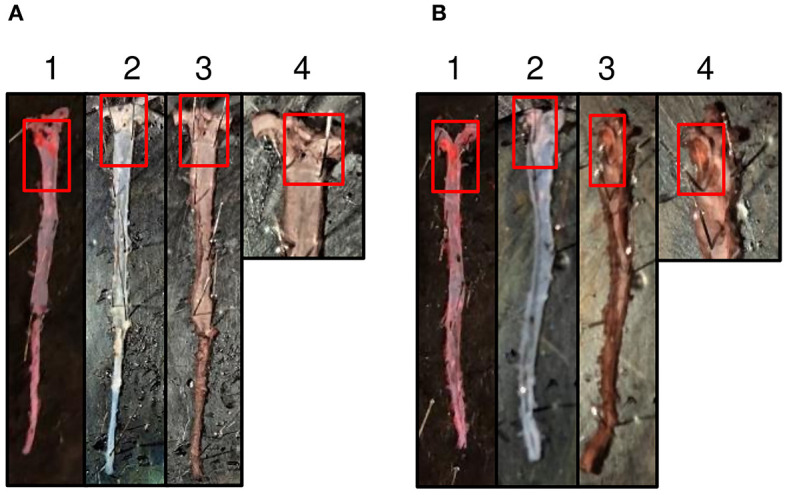
Immunostaining of mutant CRP in the aorta. **(A)** A representative aorta from untreated mice stained for mutant CRP. **(B)** A representative aorta from mice treated with mutant CRP for 7 weeks, stained for CRP. 1–4 represent an aorta through different stages of staining. (1) Sudan IV-stained aorta. Red colored areas are the lesions. (2) Aorta post-dehydration to remove Sudan IV. White colored areas are the lesion areas. (3) Anti-human CRP antibody-stained aorta. Brown colored areas reflect the presence of mutant CRP in the lesions. (4) Magnified aortic arch area of the anti-human CRP antibody-stained aorta. Red boxes show the lesion areas.

Immunostaining of CRP in the aorta isolated from mice treated with mutant CRP for seven weeks was performed to confirm the presence of administered mutant CRP at the *en face* atherosclerotic lesions in mutant CRP-treated mice. As shown (Figure 5), the lesions in the aorta of mutant CRP-treated mice were stained while the lesions in the aorta of untreated mice did not stain for CRP. The polyclonal anti-human CRP antibody used in this study does not react with purified murine CRP (data not shown), consistent with the previously published report that anti-human CRP antibodies do not react with murine CRP (30).

### Mutant CRP Does Not Alter the Concentration of Lipoproteins in the Plasma

Injecting a total of 1.6 mg of mutant CRP into mouse, in 32 injections over a period of 9 weeks, did not alter the concentration of lipoproteins in the circulation. As shown in Table 1, the concentrations of LDL and HDL in the plasma of untreated and mutant CRP-treated mice were comparable. There was no statistically significant difference in the levels of either LDL or HDL in the plasma at any time point between untreated and mutant CRP-treated groups of mice. Administration of mutant CRP did not affect the body weight. Body weights of untreated and mutant CRP-treated mice were similar at each time point (data not shown).

**Table 1:** Concentration of lipoproteins in the plasma.

**Week of sacrifice**	**HDL (mg/dl)**		**LDL (mg/dl)**	
	**Untreated**	**CRP-treated**	**Untreated**	**CRP-treated**
1	61 ± 2	85 ± 19	864 ± 16	713 ± 31
3	91 ± 15	71 ± 19	838 ± 231	786 ± 246
5	78 ± 4	74 ± 12	754 ± 118	834 ± 185
7	131 ± 1	107 ± 15	932 ± 86	*1, 086*±23
9	80 ± 3	105 ± 2	*1, 054*±3	*1, 103*±19

*Plasma HDL and LDL levels were analyzed in samples pooled from all 12 mice in each group. Results are expressed as mean ± SEM*.

## Discussion

In this study, we investigated the effects of mutant CRP F66A/T76Y/E81A, capable of binding to atherogenic LDL but incapable of binding to PCh, on the development of atherosclerosis in male *ldlr*^−/−^ mice. Our major finding was that the administration of mutant CRP for 7 weeks slowed the progression of atherosclerosis. The total size of atherosclerotic lesions in the whole aorta of mice treated with mutant CRP on alternate days for 7 weeks and beyond was significantly smaller (~40%) than the lesions in the aorta of untreated mice.

Previously, the role of WT CRP in atherosclerosis had been explored, employing a variety of experimental strategies, and 12 papers have been published using CRP from man, mouse and rabbit in both murine and rabbit models of atherosclerosis (31–42). In these studies, both, normal mice and CRP-deficient mice have been employed. Both, normal rabbits and rabbits in which CRP was inhibited by using anti-sense technology have been employed. Both, passively administered CRP and transgenic human CRP have been employed. Three different types of atherosclerosis-prone mice, *apoE*^−/−^, *ldlr*^−/−^, and *apoB*^*100/100*^ *ldlr*^−/−^, have been used in these studies. Two of the 12 papers indicated that both human and murine CRP might be playing an atheroprotective role: human CRP was shown to slow the development of atherosclerosis in the *apoB*^*100/100*^ *ldlr*^−/−^ mice (36), and the lesion size in CRP-deficient mice on *apoE*^−/−^ or *ldlr*^−/−^ background was either equivalent or increased compared to that in normal mice (41). In the other 10 papers, regardless of the experimental strategy used, WT CRP from all species did not show any effect on the development of atherosclerosis in animals, suggesting that WT CRP is neither pro-atherogenic nor anti-atherogenic (25, 43).

The most logical explanation for the observations of zero or nominal effects of WT CRP (31–42) on atherosclerosis in animal models is that the animal models of atherosclerosis do not possess the required inflammatory microenvironment that is needed by CRP to change its structure and be able to interact with atherogenic LDL (10, 18, 25). WT CRP shows no effect because pH near the lesions may not be acidic in animal models and, therefore, the structure of administered or endogenous WT CRP remains unchanged (10, 18, 25). WT CRP treated with acidic pH *in vitro* was unsuitable for administration into blood circulation of animals since the acidic pH-induced conformational alteration in the pentameric structure of CRP was found to be reversible at physiological pH (17). Therefore, we hypothesized that an *in vitro*-generated mutant CRP, capable of binding to atherogenic LDL without the requirement of any structural modification *in vivo*, would be suitable to investigate the mechanism of action of CRP on the development of the disease in animal models (10, 16, 18, 25). Indeed, in one study, monomeric CRP, that is also capable of binding to atherogenic LDL, was employed and the results showed that monomeric CRP was protective against atherosclerosis in *apoE*^−/−^ mice (34). Thus, our current findings using mutant CRP and previous findings using monomeric CRP, both molecules capable of binding to atherogenic LDL, indicate that structurally altered CRP protects against atherosclerosis; it is just that WT CRP does not exert a protective effect in most animal models (25, 44).

The effect of mutant CRP on the size of atherosclerotic lesions in the *en face* aorta was obvious since we found that administered mutant CRP had reached the aorta. The staining of aortic roots for the presence of administered mutant CRP provided inconclusive results (data not shown). It is assumed that if mutant CRP reached the aorta, it also reached the aortic root, and if this assumption is correct, then the effect of mutant CRP was site-specific. Site-specific effects of experimental manipulations have been observed in other studies using *ldlr*^−/−^ mice where the disease developed differently at various lesion-prone sites (45–50). Since the effects of mutant CRP were still observed after week 9 in the *en face* aorta, it is unlikely that anti-CRP antibodies were produced in response to intravenous administration of mutant CRP that could have inhibited its functions.

The topology of the LDL-binding site and the number of LDL-binding sites on mutant CRP remain undefined. Two possible mechanisms have been proposed for the interaction between conformationally altered CRP and atherogenic LDL. The intrinsically disordered region present in CRP has been shown to participate in the binding of monomeric CRP and atherogenic LDL (51). We proposed that the loosening of the CRP pentamer contributed to the formation of the LDL-binding site (16–18, 25). Recently, it has been suggested that the pentameric assembly of CRP harbors a pronounced plasticity in inter-subunit interactions, which may form the basis for a reversible activation of CRP in inflammation (52). However, it is unknown whether the intrinsically disordered region was exposed or the pentamer was loosened in mutant CRP employed in this study. We speculate that there is only one LDL-binding site per CRP pentamer. However, for the protection of mice against atherosclerosis, it is possible that both sites, the PCh-binding site and the LDL-binding site, participate. The search for a new mutant CRP which can bind to both PCh and to atherogenic LDL is in progress. Any possible similarity between mutant CRP used in this study and previously reported conformationally altered pentameric forms of CRP, pCRP^*^, and mCRPm (20, 21), is unknown.

Based on the data obtained from a single regimen for mutant CRP treatment, we conclude that one of the functions of CRP is to confer protection against atherosclerosis. We propose, again, that there is no need to stop the biosynthesis of CRP, and that a drug that can lower cholesterol level but not CRP levels could be superior to statins which reduce both CRP and cholesterol levels (53, 54). These suggestions are supported by the fact that, in rabbits, the inhibition of plasma CRP did not affect the development of atherosclerosis (42). It has also been previously postulated that the deposition of CRP at the atherosclerotic lesions may be independent of the CRP levels in the circulation and that CRP-mediated lipoprotein removal likely underlies the regression of early lesions which occurs continuously throughout life and that CRP should be considered as an anti-atherosclerotic protein (10, 13). A long-term goal should be the discovery and design of small-molecule compounds to aid endogenous native CRP in capturing atherogenic LDL, as proposed earlier (14, 25). Another goal could be to investigate the possible protective effects of mutant CRP used in this study in animal models of other inflammatory diseases.

Our data also provide a proof of concept that a local inflammation-induced structural change in native CRP is a prerequisite for CRP to control the development of atherosclerosis. An appropriate inflammatory microenvironment at the site of LDL deposition seems to be critical for CRP to prevent atherosclerosis. One function of inflammation could be to change the structure of proteins, including CRP. Inflammation is not a silent killer, perhaps, as has been suggested (55).

## Data Availability Statement

All datasets generated for this study are included in the article/Supplementary Material.

## Ethics Statement

The animal study was reviewed and approved by University Committee on Animal Care, East Tennessee State University.

## Author Contributions

AP, SS, and DT performed the experiments. AP and AA analyzed the data. AA conceived and designed the experiments and wrote the paper. All authors contributed to the article and approved the submitted version.

## Conflict of Interest

The authors declare that the research was conducted in the absence of any commercial or financial relationships that could be construed as a potential conflict of interest.

## Abbreviations

CRP: C-reactive protein
LDL: Low-density lipoprotein
*Ldlr*^−/−^: LDL receptor-deficient
PCh: Phosphocholine
WT: wild-type.
