# Supplementary figures and images for: Conformationally Altered C-Reactive Protein Capable of Binding to Atherogenic Lipoproteins Reduces Atherosclerosis
Source: Front Immunol. 2020 Aug 11;11:1780. doi: 10.3389/fimmu.2020.01780 (PMC7431523; doi:10.3389/fimmu.2020.01780)

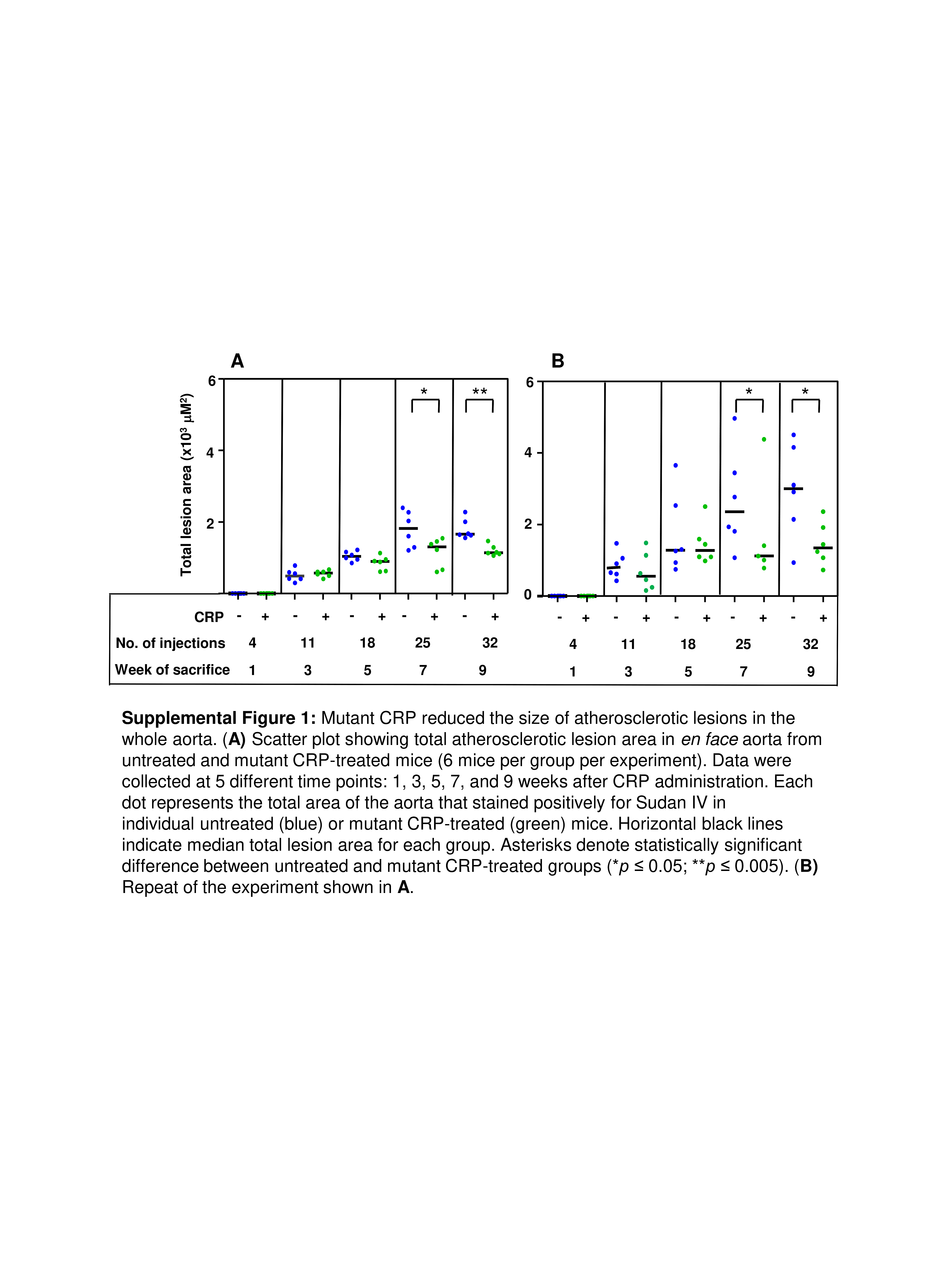

Supplement: Supplementary file 1 [file Image_1.JPEG]

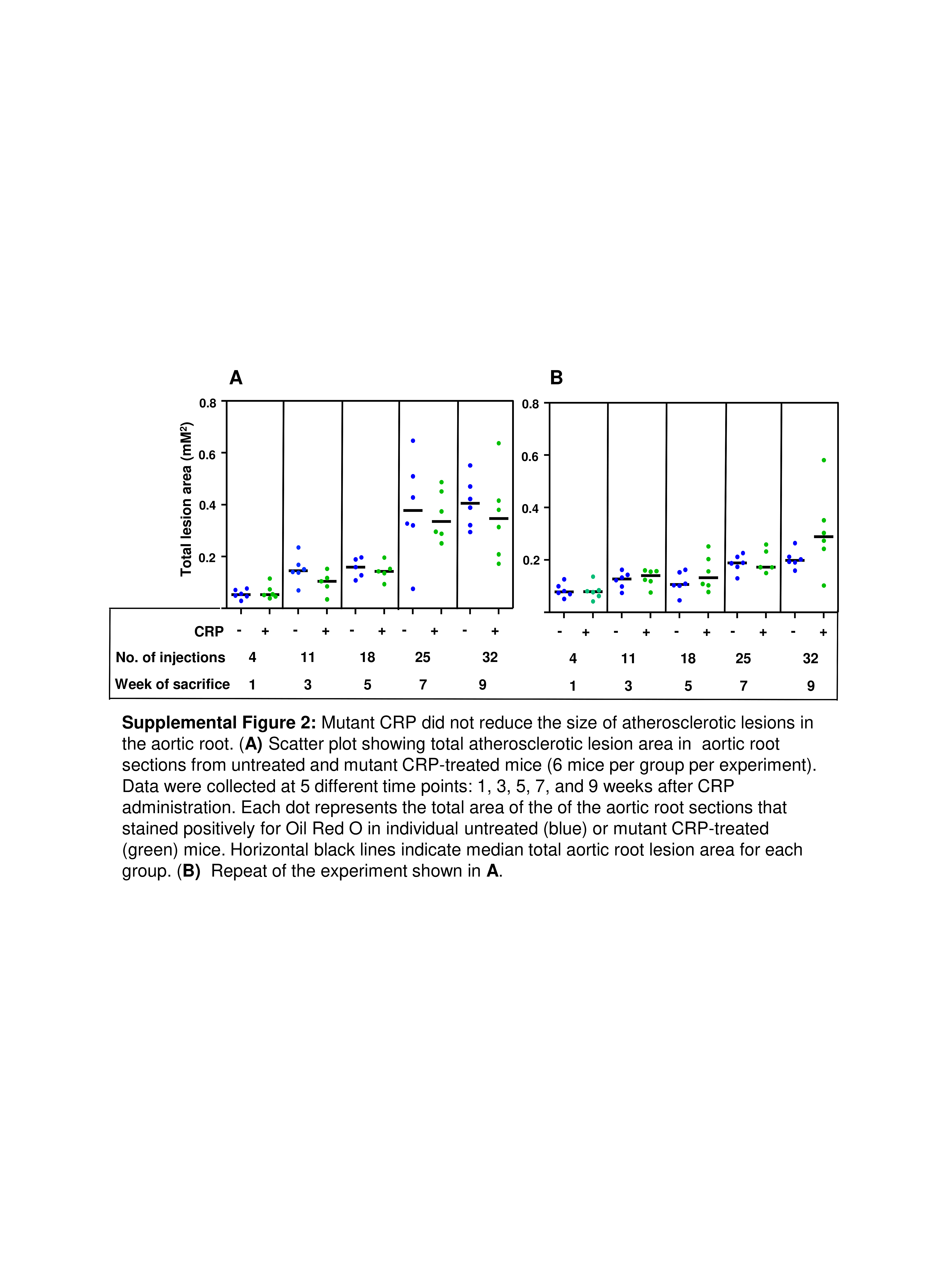

Supplement: Supplementary file 2 [file Image_2.JPEG]
